# Supplementary figures and images for: Interplay between Intestinal Bacterial Communities and Unicellular Parasites in a Morbidly Obese Population: A Neglected Trinomial
Source: Nutrients. 2022 Aug 5;14(15):3211. doi: 10.3390/nu14153211 (PMC9370494; doi:10.3390/nu14153211)

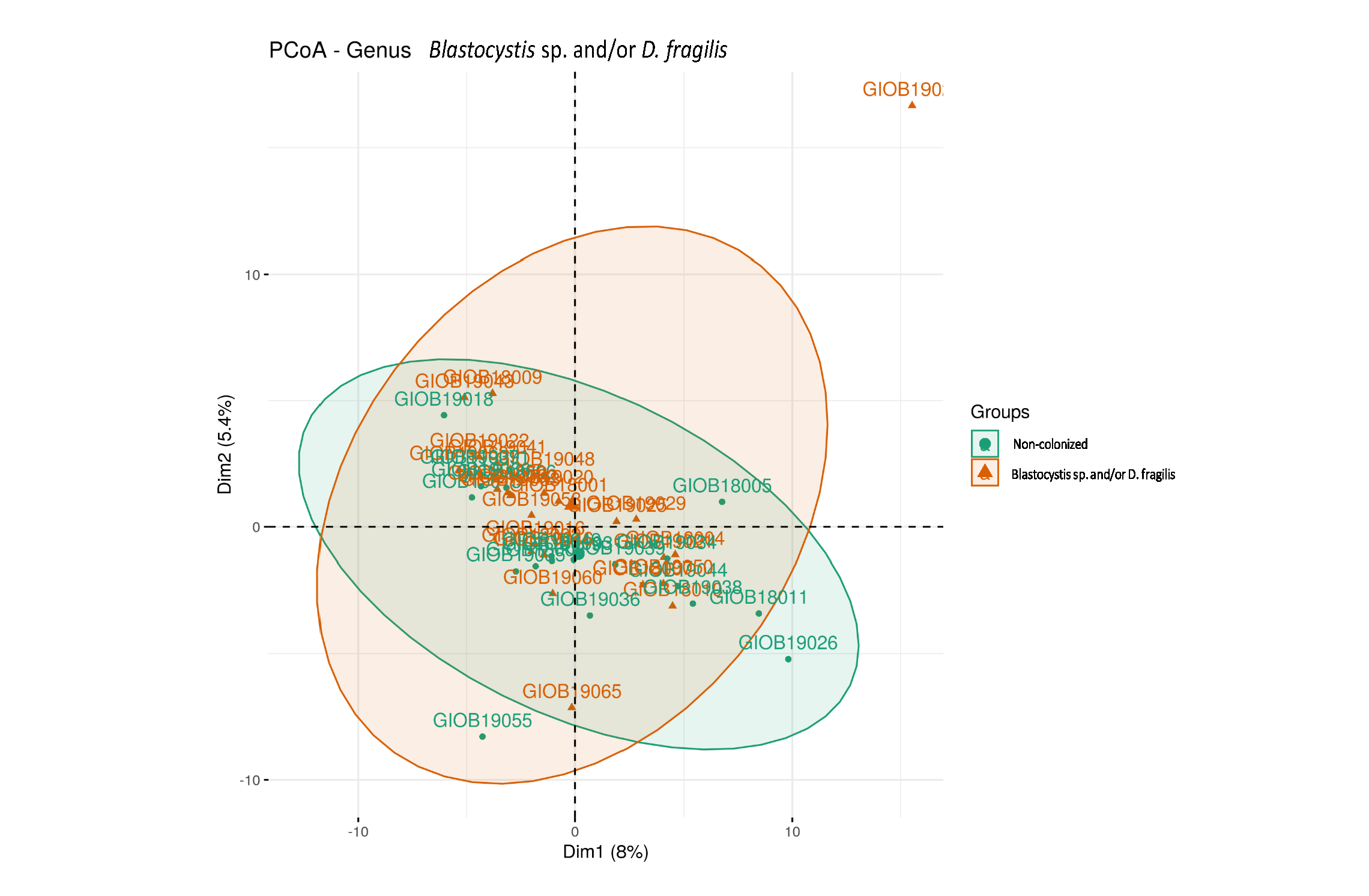

Supplement: Supplementary file 1 [file nutrients-14-03211-s001.zip › Figure S1 PCoA genus plots clusered by colonization status by Blastocystis sp. Dientamoeba fragilis.tiff]

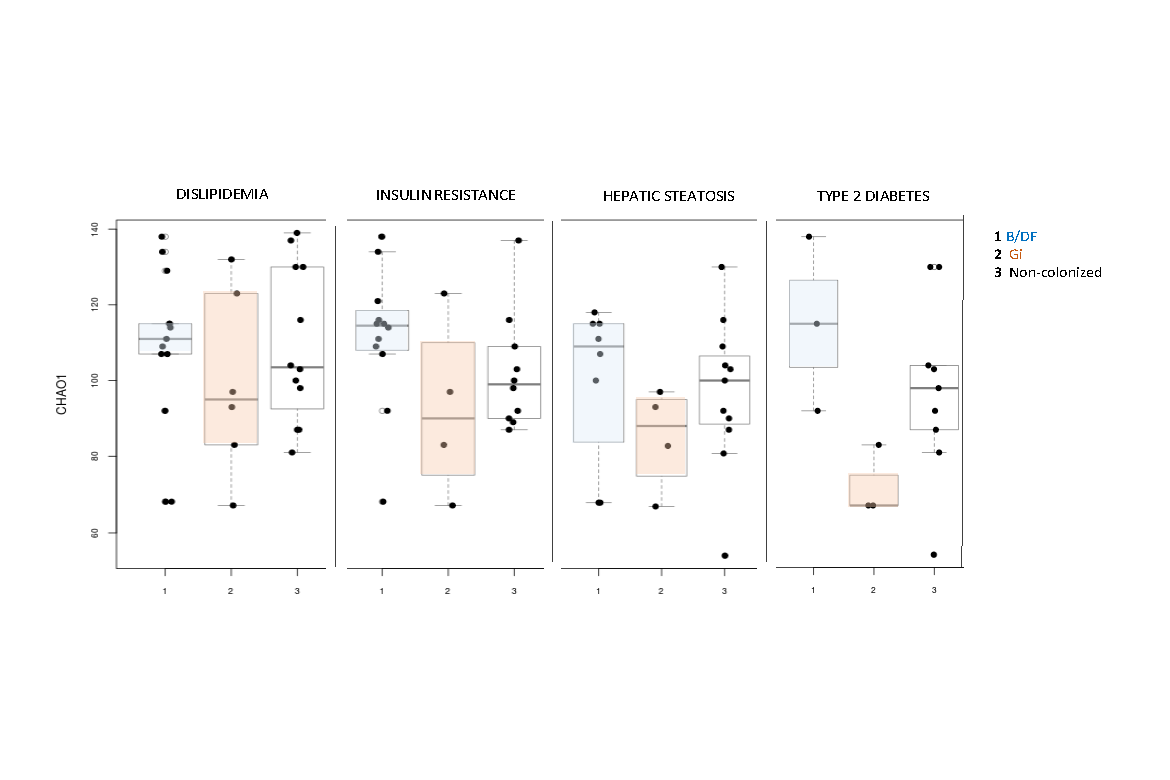

Supplement: Supplementary file 1 [file nutrients-14-03211-s001.zip › Figure S2 Alpha Diversity in patients with metabolic comorbidities clustered by colonization status.tiff]

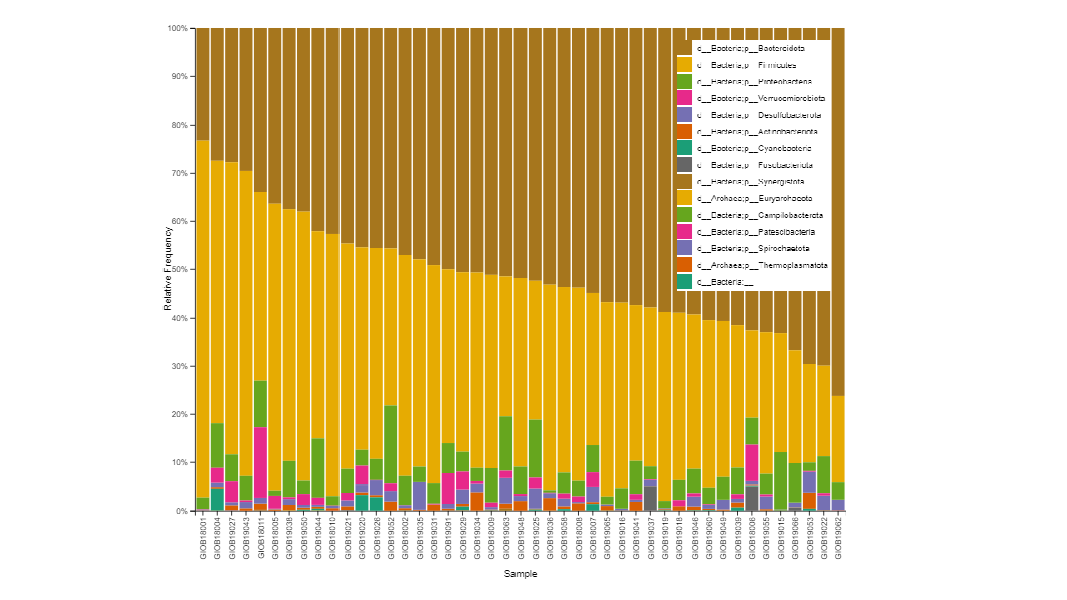

Supplement: Supplementary file 1 [file nutrients-14-03211-s001.zip › Figure S3 Relative Abundance of Phyla in studied population.tiff]

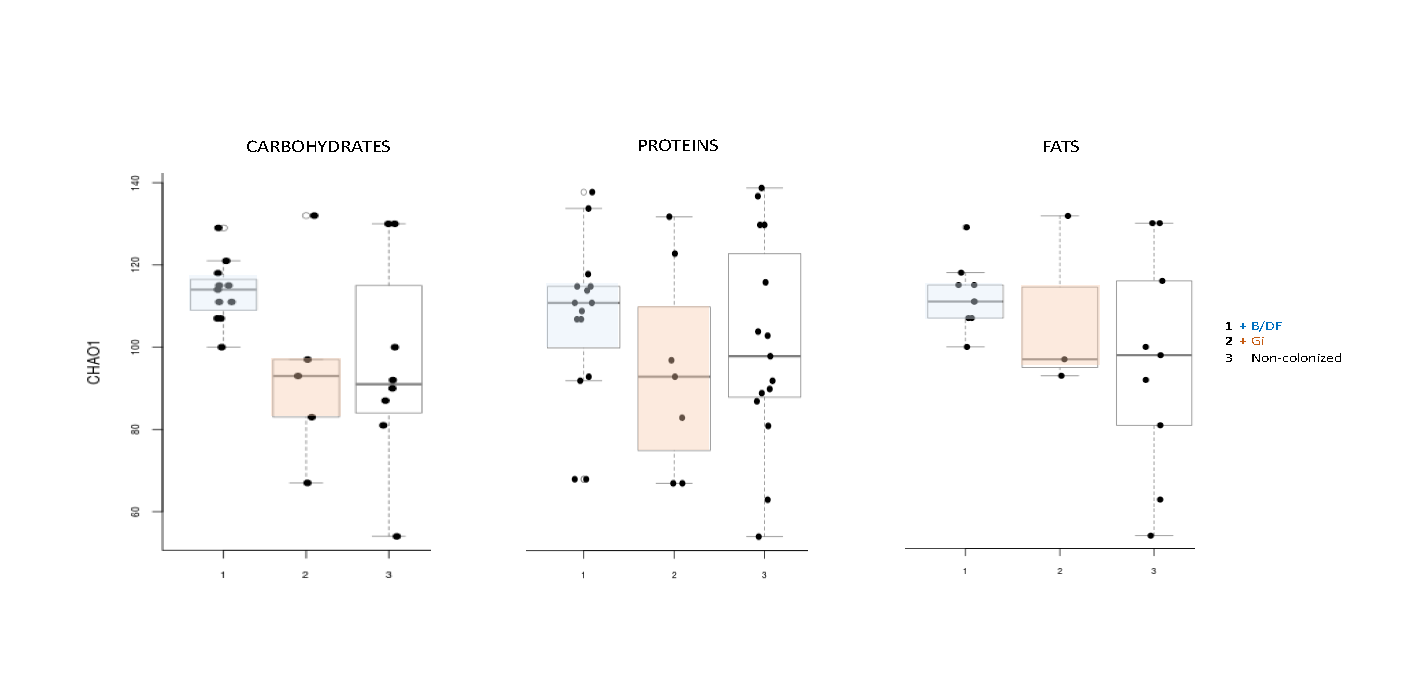

Supplement: Supplementary file 1 [file nutrients-14-03211-s001.zip › Figure S4 Alpha Diversity clustered by correct macronutrients intake and colonization status.tiff]
